# Validation of Pasteurisation Temperatures for a Tomato–Oil Homogenate (*salmorejo*) Processed by Radiofrequency or Conventional Continuous Heating

**DOI:** 10.3390/foods12152837

**Published:** 2023-07-26

**Authors:** Marina Kravets, Cristina Cedeño-Pinos, Andrés Abea, Maria Dolors Guàrdia, Israel Muñoz, Sancho Bañón

**Affiliations:** 1 Department of Food Technology and Science and Nutrition, Veterinary Faculty, Regional Campus of International Excellence “Campus Mare Nostrum”, University of Murcia, 30100 Murcia, Spain; marina.kravets@um.es (M.K.); cristinacarmen.cedenop@um.es (C.C.-P.); 2 Institut de Recerca i Tecnologia Agroalimentàries IRTA-Food Technology Program, Finca Camps i Armet, Monells, 17121 Girona, Spain; andres.abea@irta.cat (A.A.); dolors.guardia@irta.cat (M.D.G.); israel.munoz@irta.cat (I.M.)

**Keywords:** fresh-like, dielectric heating, microbiological and enzyme inactivation, thermal damage

## Abstract

Salmorejo is a viscous homogenate based on tomato, olive oil and breadcrumbs commercialised as a “fresh-like” pasteurised–chilled purée. Due to its penetration, dielectric heating by radiofrequency (RF) might improve pasteurisation results of conventional heating (CH). The objective was to validate the pasteurisation temperature (70–100 °C, at 5 °C intervals) for salmorejo processed by RF (operating at 27.12 MHz for 9.08 s) or conventional (for 10.9 s) continuous heating. The main heat-induced changes include: orangeness, flavour homogenisation, loss of freshness, thickening, loss of vitamin C and lipid oxidation. Both CH and RF equivalent treatments allowed a strong reduction of total and sporulated mesophilic microorganisms and an adequate inhibition of the pectin methylesterase, peroxidase and, to a lesser extent, polyphenol oxidase but did not inhibit the polygalacturonase enzyme. Pasteurisation at 80 °C provided a good equilibrium in levels of microbiological and enzymatic inhibition and thermal damage to the product. Increasing this temperature does not improve enzyme inactivation levels and salmorejo may become overheated. A “fresh-like” good-quality salmorejo can be obtained using either conventional or radiofrequency pasteurisers.

## 1. Introduction

Tomato-based homogenates are traditional Spanish dishes and an essential part of the Mediterranean diet which contribute to fruit and vegetable intake. Salmorejo is a cold purée prepared with tomato, olive oil, bread, garlic, vinegar and salt. Traditionally, salmorejo has been a raw, home-made product especially consumed in the summer months. In recent years, there has been increasing industrial production of “fresh-like” salmorejo (about 26 million litres in 2021) and its consumption is spreading in Spain and other European countries [1]. “Fresh-like” salmorejo maintains most of the sensory and nutritional characteristics of the product with no preservation treatment (similar to homemade), being appreciated by the consumers of minimally processed food. Salmorejo is manufactured in the same industrial lines used for gazpacho (a similar tomato product prepared with cucumber, onion or pepper) and is often pasteurised by conventional heating (CH) using heat exchangers. Pasteurisation conditions are similar to those for gazpacho: from 70 °C for 30 s (fresh-like product stable at 4–10 °C up to 2 months) to 92–95 °C for 1 min (stable at room temperature up to 12 months) [2]. This extended shelf life can be achieved as the natural acidity of tomato helps to inhibit microbial growth [3]. However, pasteurisation of salmorejo presents some difficulties: (i) it can reach a semi-solid state that may limit its processing in conventional heat exchangers; (ii) it is a product prone to sensory alteration by heating (e.g., discolouration, cooked off-flavours and thickening); and (iii) mild heating may not adequately inactivate the tomato enzymes responsible for its clarification (pectin methylesterase “PME” and polygalacturonase “PG”) and oxidative deterioration (polyphenol oxidase “PPO” and peroxidase “POD”) [4,5,6,7]. Thus, the use of more efficient preservation methods that improve stabilisation of “fresh-like” salmorejo could help to overcome the above limitations.

Alternative heating technologies used to pasteurise “fresh-like” tomato products include: microwave (MW) continuous heating (2450 W; 82 s) vs. equivalent CH (85 °C; 5 min) in tomato juice [8]; MW heating (390–3150 W; 150–848 s) vs. equivalent CH (96 °C; 35 s) in tomato purée [9]; MW heating (950 W; 3 min) vs. CH (93.3 °C; 26.5 min) in tomato purée [10]; MW heating (210–3600 W; 93–646 s) vs. CH (90 °C; 35 s) in tomato smoothie [11]; and ohmic heating (90 °C; 15–60 s) vs. CH (90 °C; 75–300 s) in concentrated tomato paste [12]. Moreover, some attempts with non-thermal technologies such as high pressures [13] and pulse electric fields (50–450 Hz, 1–4 pulses) [6,14,15] have also been conducted in gazpacho, juices and tomato smoothies. From these studies, focused on partial aspects (microbial and enzyme inactivation, retention of nutrients, volatile compounds or sensory traits), it can be stated that stabilisation obtained by CH can be reached or even improved using other technologies. Among technologies available at the industrial scale, dielectric heating by radiofrequency (RF) has not yet been tested to process “fresh-like” products such as salmorejo. In dielectric heating, the energy is transferred through electromagnetic waves into the food material, reducing overheating and temperature differences within the product. RF heating works at lower frequencies (10–300 MHz) than MW heating (300 MHz–300 GHz). Compared to MW, RF allows the wave energy to penetrate deeper into the product, the heating pattern is more uniform and less overheating is caused when raising the temperature of the cold spots to the target values. Therefore, RF heating shows potential to improve the quality of thermally treated products and to avoid fouling at heat-transferring surfaces [16]. As RF heating reduces temperature differences in the product, this technology might be suitable for pasteurising high-viscosity fluids [17], overcoming the low rate of heat transfer in conventional thermal processing caused by the difficulty of mixing fluid layers of the product. Another advantage is the high efficiency of energy transfer from energy source to the product (over 80%), with lower energy losses to the environment [18]. The reduced fouling deposition due to the application of energy directly to the product is also noteworthy, since it is one of the main problems in tubular exchangers, decreasing system efficiency and requiring large amounts of water and cleaning agents [16].

In the scientific literature, food applications of dielectric heating are dominated by MW [19]. The implications of using MW to process fluid foods (microbial and enzyme inactivation, chemical, physical or sensory changes or reheating) have been widely studied [20]. The major drawback associated with MW heating is the non-uniform temperature distribution, resulting in hot and cold spots in the heated product, which may affect the inactivation of microorganisms and enzymes [21]. MW technology has been applied to different liquid products: green pea/carrot purée, fruit juices, purées and sauces. RF technology is not so commonly used as MW but has also been applied to liquid foods such as kiwi purée [22], yoghurt [23], liquid egg [24] and fish soup [25]. RF heating has been proposed as a technology to inactivate enzymes present in foods. RF proved efficient in inactivating POD enzyme in lettuce, reducing thermal damage compared to CH [26] although there is little information on other vegetable products. It was reported that MW heating is as efficient as CH to inactivate POD, PPO and PG tomato enzymes [9,11,27], therefore these findings can also be achieved using RF treatments. In other vegetable products, a higher degree of enzyme inactivation was found for CH in grapefruit [28] and strawberry purée [29], whereas MW proved more effective than CH in orange juice [30]. RF technology could replace conventional heat exchangers in the food industry as dielectric heating would be less energy-intensive [31]. 

A preliminary trial addressed how dielectric properties are affected by the temperature and oil and salt contents in tomato–oil emulsions [32]. In another recent study, RF heating of packed tomato purée moving on a conveyor belt was modelled [33]. The next challenge is to develop pasteurisation methods with RF technology that can be implemented in the industry. The research hypothesis was that RF might be more effective than CH in stabilising a viscous thermolabile product like salmorejo when equivalent high-temperature short-time (HTST) pasteurisation conditions (in terms of microbial inactivation) are applied. The objective was to establish the optimal temperature (70–100 °C, at 5 °C intervals) for pasteurising salmorejo by radiofrequency or conventional continuous heating.

## 2. Materials and Methods

### 2.1. Experimental Design

A randomised factorial design with the heating method and pasteurisation temperature as main treatments was performed for the experiment. Three batches of salmorejo were alternately pasteurised in a conventional heat exchanger or in RF equipment applying a temperature range of 70 to 100 °C at intervals of 5 °C. Sample size was *n* = 126 (3 production batches × 3 salmorejo bottles × 2 heating methods × 7 temperatures), excluding raw samples from each batch. All samples were analysed in triplicate. 

### 2.2. Salmorejo Ingredients and Manufacturing Process

An experimental formulation for salmorejo with a partial substitution of breadcrumbs for a high-viscosity inulin was developed for the project. Salmorejo ingredients were purchased from a local supermarket. Orafti^®^ high-polymerisate (HP) inulin was provided by Beneo Ibérica (Barcelona, Spain). Raw product was formulated (g/100 g) as follows: vine tomato (87.6), extra virgin olive oil (Var. Hojiblanca) (5.0), breadcrumbs (3.0), inulin (2.0), wine vinegar (1.5), sodium chloride (0.7) and garlic powder (0.2).

**Figure 1:**
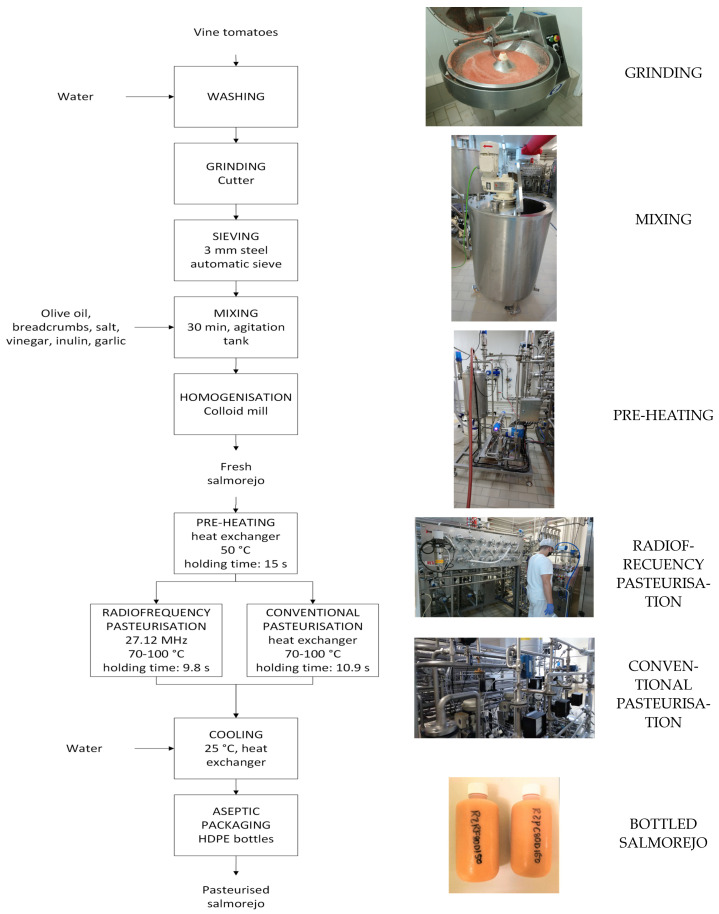
Flow diagram for elaboration for salmorejo pasteurised by conventional or radiofrequency heating.

The manufacturing process of salmorejo is shown in Figure 1. Vine tomatoes were washed, ground in a cutter and placed in a 3 mm steel automatic sieve. The resulting tomato purée was then mixed with olive oil, breadcrumbs, salt, vinegar, inulin and garlic for 30 min in an agitation tank. Raw purée was homogenised in an MZ-100 colloid mill (FrymaKoruma AG, Rheinfelden, Switzerland) to obtain raw salmorejo. This was alternatively pasteurised using continuous-flow CH or RF technology. The equipment could process 200 L/h of product. CH was conducted in a 2-stage tubular heat exchanger built by Inoxpa (Banyoles, Barcelona, Spain) with a holding time of 10.9 s. For RF, salmorejo was pasteurised using a 45 kW EVO RF (Cartigiliano S.p.A, Vicenza, Italy) working at 27.12 MHz with a holding time of 9.08 s and a maximum temperature increment of 50 °C. Holding times were different for RF and CH due to slight variations in the length of the holding tubes. Both processes share the first stage of the tubular heat exchanger as a pre-heating step (50 °C for 15 s). The product then passes through a high-pressure homogeniser (HA31002, Bertoli SRL, Parma, Italy), working at 250 bar, before the second processing stage (2nd tubular heat exchanger or RF equipment) to prevent coalescence of oil droplets during storage. This system allows the pasteuriser to reduce the heating ramp, therefore taking a shorter time to heat the salmorejo. After processing, the product was cooled to 25 °C using a heat tubular exchanger and aseptically packaged in 250 mL plastic (high-density polyethylene) bottles. The salmorejo was kept frozen at −18 °C until further analysis.

### 2.3. Microbiological Analyses

Total mesophilic aerobic (TMA) microorganisms were enumerated on plate count agar (PCA, Scharlab, Barcelona, Spain) after incubation at 30 °C for 48–72 h [34]. Mesophilic spores were enumerated after applying a heat treatment at 80 °C for 10 min and plating on PCA and incubated at 30 °C for 24–48 h [35]. The detection limit was 1 Log CFU g^−1^. The inactivation (Log reduction) of TMA and spores was quantified as the difference between the counts (Log CFU g^−1^) of samples before and after treatments.

### 2.4. Pasteurisation Conditions

The thermal treatment was targeted to inactivate *Escherichia coli* O157:H7, *Salmonella enterica* and *Listeria monocytogenes* to ensure at least a 5-Log reduction of vegetative pathogens of concern. As salmorejo mainly contains tomato, data corresponding to an unacidified tomato purée at 4.5 pH were used for the determination of the processing temperature ^(1)^. A minimum cumulative total lethality equivalent to *p* = 0.51 min at 71.1 °C was needed for a cocktail of pathogens [36].

(1) $$P=\int_{t=0}^{t=t_{total}}10^{\left(\frac{T-T_{ref}}{Z}\right)}dt$$

where: *P* is the pasteurisation value (min), *T* is the treatment temperature (°C), *T_ref_* is the reference temperature (71.1 °C) at which thermal resistance (*Z*  =  7.37 °C) has been determined and *t* is the holding time after reaching treatment temperature (min) as described in [36]. The holding time was calculated from the flow rate of salmorejo, diameter and length of tube and the flow regime (turbulent or laminar). In this setting, a laminar flow was considered based on the Reynolds number. In accordance with the formula, the set-point temperature for RF and CH heating was approximately 79.5 °C.

### 2.5. Enzyme Relative Activities

Pectin methylesterase (PME) relative activity with respect to the untreated product (%RE) was determined according to the method of Arjmandi et al. (2017a) [11] with slight modifications. First, 2.5 mL of salmorejo was mixed with 10 mL of 0.2 M NaCl solution. This was filtered with cotton to remove the fraction not soluble in water. Then, 2.5 mL of filtrate was mixed with 15 mL of a 1% (*w*/*v*) citrus pectin solution containing 0.2 M NaCl solution. The pH was adjusted to 7.0 using some drops of 0.2 M NaOH solution. The sample was titrated with 0.01 M NaOH solution for 10 min using a GLP21 pH meter (Crison, Barcelona, Spain). PME activity (expressed as PME units mL^−1^) was calculated using the following Formula (2):


(2) $$PME\;units\;{\mathrm{mL}}^{-1}\;=\;\frac{\mathrm{mL}\;NaOH\;*\;normality\;of\;NaOH\;}{\mathrm{mL}\;sample\;\left(g\right)\times 10\;\min}\times 1000$$

Polygalacturonase (PG) %RE was determined according to Fachin et al. (2003) [37]. First, 3 mL of salmorejo was mixed with 3 mL of acidified water (37% HCl; pH 3) at 4 °C in an RX3 vortex mixer (Velp Scientifica™, Usmate Velate MB, Italy). The mix was kept under agitation in a Rotavit (JP Selecta™, Barcelona, Spain) at 170 u/min, 4 °C and darkness for 20 min. The sample was centrifuged in a Digicen 21 R centrifuge (Orto Alresa, Madrid, Spain) at 4554× *g* and 4 °C for 10 min. Sample precipitate was reconstituted until reaching 6 mL with a 1.2 M NaCl solution, shaken (at 170 u/min and 4 °C for 1 h) and centrifuged (at 4554× *g* and 4 °C for 10 min) again. The supernatant was filtered through Whatman No. 1 paper to obtain enzyme extract. Then, l00 µL of enzyme extract was mixed with 300 µL of 0.2% (*w*:*v*) polygalacturonic acid (Sigma, St. Louis, MO, USA) and incubated in a water bath at 35 °C and darkness for 10 min. To stop the reaction, the sample was first mixed with 2000 µL of 0.1 M borate buffer at pH 9 (composed of Na_2_B_4_O_7_ 10H_2_O and H_3_BO_3_ at 1.47/0.38 *w*:*w*) and 400 µL of 1% (*w*:*v*) cyanoacetamide as substrate and then incubated in a water bath at 100 °C for 10 min. The sample was cooled at room temperature, transferred to 3.5 mL quartz cuvettes and measured at 276 nm in a UV–Vis Genesys™ 180 spectrophotometer (Thermo Fisher Scientific, Madison, WI, USA). The enzyme extract was replaced by distilled water to obtain the blank.

Peroxidase (POD) %RE was determined according to Vervoort et al. (2012) [38]. First, 3 mL of salmorejo was mixed with 6 mL of 0.2 M sodium phosphate buffer (pH 6.5) and centrifuged at 4554× *g* and 4 °C for 15 min. The sample was filtered with a Whatman No. 1 paper to obtain enzyme extract. Then, 0.25 mL of enzyme extract was mixed with 1.1 mL of 0.2 M sodium phosphate buffer (pH 6.5), 0.5 mL of o-phenylenediamine solution (10 g L^−1^ in 0.2 M sodium phosphate buffer pH 6.5) as substrate and 0.25 mL of hydrogen peroxide solution (15 g L^−1^ in 0.2 M sodium phosphate buffer pH 6.5) as oxidising reagent. The sample was kept at 25 °C and in darkness for 20 min and absorbance was then measured at 485 nm. The enzyme extract was replaced by distilled water to obtain the blank.

Polyphenol oxidase (PPO) %RE was assessed according to Marszałek et al. (2015) [29]. First, 3 g of salmorejo was mixed with 6 mL of 0.2 M sodium phosphate buffer (pH 7.0) containing 10 g of L^−1^ insoluble polyvinylpyrrolidone and 5 g of L^−1^ Triton X-100. The mix was centrifuged at 4554× *g* and 4 °C for 30 min. The sample was filtered with a Whatman No. 1 paper to obtain enzyme extract. Then, 75 µL of enzyme extract was mixed with 1 mL of 0.21 M catechol solution (0.05 M sodium phosphate buffer: pH 6.5) as substrate and 2 mL of 0.2 M sodium phosphate buffer and measured at 420 nm and 25 °C. The enzyme extract was replaced by distilled water to obtain the blank. PPO activity was calculated using a linear regression obtained from the absorbance values measured every min for 30 min. 

### 2.6. Physicochemical Analyses

Ascorbic acid (mg 100 mL^−1^) was determined according to Sánchez-Mata et al. (2000) [39]. First, 3 g of salmorejo was added with HPO_3_ 4.5% (*w*/*v*) water solution until reaching 10 mL in a volumetric flask. The mixture was stirred for 1 min and then centrifuged at 4053× *g* and 4 °C for 10 min. The supernatant was filtered with a Whatman N°1 filter to obtain sample extract. Then, 1.5 mL of sample extract was filtered with a 0.45 µm Millipore SAS filter and injected into an Infinity II HPLC/DAD-1260 Series system (Agilent Technologies, Santa Clara, CA, USA). A Brisa LC2C18 column (25 × 0.46 cm) with 5 µm pore size was used (Teknokroma, Barcelona, Spain). Operating conditions were: (i) flow rate: 0.8 mL min^−1^; (ii) mobile phase: acidified water (2.5% sulphuric acid *v*/*v*); (iii) temperature: 35 °C; (iv) analysis time duration: 20 min; and (v) detection wavelength: 245 nm. 

Thiobarbituric acid reagent substances (TBARSs; mg MDA Kg^−1^) were measured according to Botsoglou et al. (1994) [40]. First, 4 g of salmorejo was mixed with 8 mL of trichloroacetic acid (TCA) water solution (5% *w*/*w*) and mL of butylated hydroxy toluene (BHT) hexane solution (0.8 *w*/*v*) as antioxidant. The mix was centrifuged at 3000 rpm and 4 °C for 10 min. Approximately 8 mL of supernatant was increased to 10 mL with a TCA water solution (5% *w/v*) and centrifuged again (at 1328× *g* and 4 °C for 10 min) to improve sample turbidity. Then, 2.5 mL of supernatant was mixed with 1.5 mL of thiobarbituric acid (TBA) water solution (5% *w/v*) and incubated in a water bath at 70 °C for 30 min. Sample absorbance was measured at 532 nm and 25 °C. Sample was replaced by TBA water solution to obtain the blank. A malondialdehyde (MDA) standard calibration curve was prepared with 1,1,3,3-tetraethoxypropane (Sigma-Aldrich) 0.1M HCl water solution (*v*/*v*) at concentrations ranging from 0.1–10 μM (y = 1171x + 0.0038; R^2^ = 1).

The pH was measured with a GLP21 pHmeter (Crison, Barcelona, Spain) equipped with a combined electrode, Cat. No. 52–21 (Ingold Electrodes, Wilmington, North Carolina, USA) according to the Association of Official Analytical Chemists [41]. Instrumental colour was measured using a CR-200/08 Chroma Meter II (Minolta Ltd., Milton Keynes, UK) with a D65 illuminant, 2° observer angle and 50 mm aperture size. Results were expressed as CIELAB values: lightness (L*) redness (a*), yellowness (b*) and hue angle (tg^−1^ b*/a*). Dynamic viscosity was measured by two different procedures. Viscosity 1 (V1; mPa s) including initial shear stress was determined at 20 °C using an MV1 cylindrical probe in a Haake 550 rotational viscometer (Thermo Scientific, Waltham, MA, USA). Flow curves were obtained from a stepped shear stress ramp (steady state approximation: 15 s per point). Ranges of shear stresses, in a linear distribution, were used to obtain shear rates between 450 and 1800 s^−1^. Flow curve data were fitted to the Bingham model (τ = τ0B + γμpl), where τ is the shear stress (Pa), γ˙ the shear rate (s^−1^), τ0B the Bingham yield stress (Pa) and μpl the plastic viscosity (Pa s). Dynamic viscosity (V2; Pa s) was also measured with a standard cylindrical vessel of 125 × 28 mm (head space, 20 mm) using a 6 L/plus rotational viscotester (Haake, Thermo Scientific, Karlsruhe, Germany). The measuring conditions for V2 were: 80 mL of sample; L3 probe; 100 rpm angular speed; 1 min at 4 °C. Texture was analysed using a QTS-25 Analyser (Brookfield, Harlow, Essex, England). Operating conditions were: 5 °C; TA3/100 flat cylindrical probe (20 mm in diameter); trigger point, 0.05 N; compression objective, 5 mm; crosshead speed, 0.1 mm/s; and charge cell, 10 Kg. Results were expressed as hardness (N), the maximum force required to compress the material, and as the deformation energy (mJ) required to deform the sample.

### 2.7. Sensory Analysis

A quantitative descriptive analysis of salmorejo samples was performed in 10 sessions/replicates. Generation of descriptors for samples was carried out by open discussion in two previous sessions. Selection of attributes included in the sensory profile was based on those that help to discriminate the tested treatments. Descriptors retained and their definition are presented in Table 1. Six trained panellists [42,43] performed sensory analysis on 50 mL of salmorejo sample using an incomplete block design. A non-structured scoring scale was used, with 0 as absence of descriptor and 10 as highest intensity of descriptor. Samples were coded with three-digit random numbers and presented to assessors, balancing the first order and the carry over effect [44]. The average scores of each sample and session were recorded and used in data analysis.

### 2.8. Statistical Analysis

The effects of treatments on the dependent variables (inactivation of bacteria and enzymes and thermal damage) were determined by two-way ANOVA. Differences among means were tested with the Tukey range test (*p* < 0.05). Enzyme activities at different temperatures were predicted using exponential and polynomial regression second order models. Data were analysed with the Statistix 8.0 software for Windows (Analytical Software, Tallahassee, FL, USA).

## 3. Results

### 3.1. Microbiological and Enzyme Inactivation

**Figure 2:**
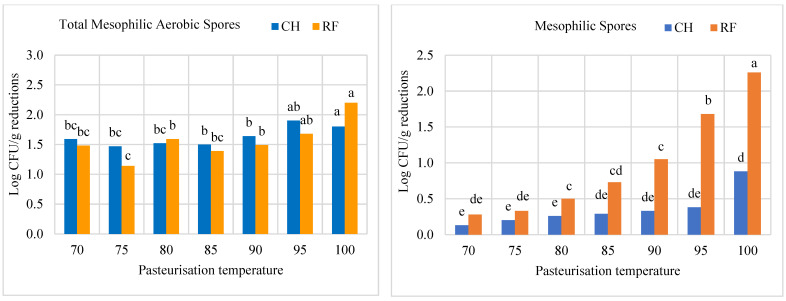
Log CFU g^−1^ reductions in counts of total mesophilic aerobic and mesophilic spores in salmorejo pasteurised by convectional (CH) or radiofrequency (RF) heating and at different temperature (70–100 °C). Root mean standard error: 0.139 (TMA) and 0.133 (spores). ^a–e^ Effects of heating method x temperature for *p* < 0.05 (Tukey test).

**Figure 3:**
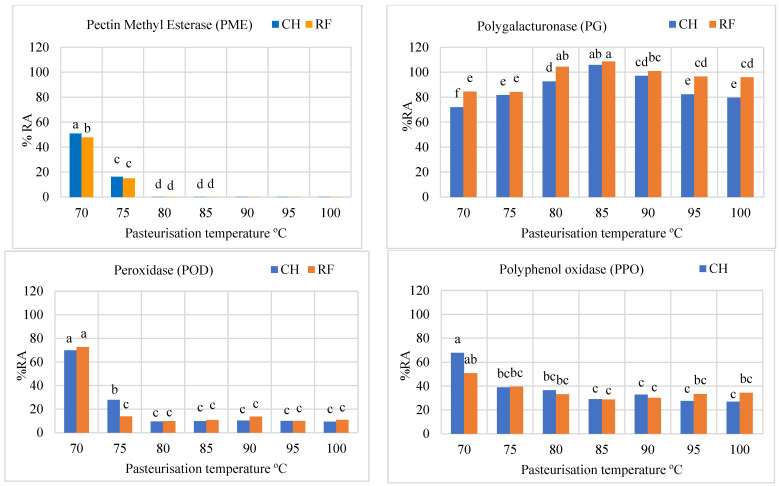
Relative enzyme activities (%RA) determined in salmorejo pasteurised by convectional (CH) or radiofrequency (RF) heating at different temperatures (70–100 °C). Root mean standard error: 1.00 (PME), 1.77 (PG), 1.42 (POD) and 4.89 (PPO). ^a–f^ Effects of heating method and temperature for *p* < 0.05 (Tukey test). There was significant (*p* < 0.05) interaction between treatments for PG, POD and PPO %RA.

**Figure 4:**
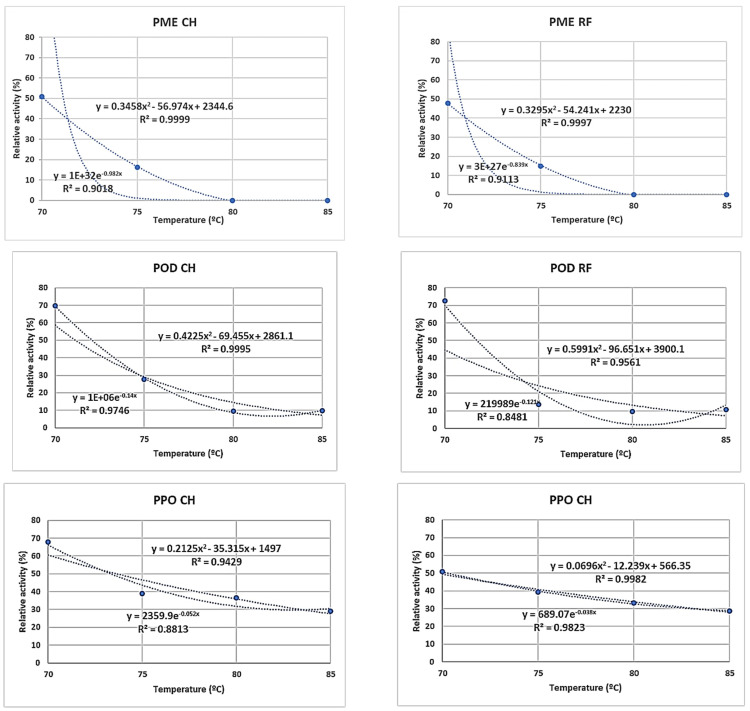
Inactivation predictive models (polynomic and exponential regressions) at 70–85 °C for pectin methylesterase (PME), peroxidase (POD) and polyphenol oxidase (PPO) enzymes in salmorejo pasteurised by convectional (CH) or radiofrequency (RF) heating.

Data on microbiological and enzyme inactivation are shown in Figure 2 and Figure 3. Counts of TMA for raw salmorejo were 4.01 (CH) and 4.23 Log CFU g^−1^ (RF). Log reductions in TMA counts were similar (*p* > 0.05) for CH (1.5–1.9) and RF (1.1–2.2) salmorejo. The highest TMA Log reduction was reached in RF samples at 100 °C. Counts of mesophilic spores for raw salmorejo were 2.60 (CH) and 3.03 Log CFU g^−1^ (RF). Log reductions in counts of mesophilic spores clearly tended to increase with temperature, particularly in RF samples. RF was more efficient than CH in inactivating mesophilic spores from 90 °C onwards. As regards enzymes, PME was completely inactivated at temperatures from 80 °C onwards in CH and RF samples. PG showed over 80–90% RE or even higher (intermediate temperatures) for most treatments; CH proved more efficient than RF in inhibiting this enzyme at 70 °C and at 95–100 °C. POD was strongly inactivated (<10% RE) from 80 °C onwards, while PPO showed over 30% RE in CH and RF samples at 75–100 °C. Tomato enzymes were classified according to their thermal resistance as follows: PG > PPO > POD > PME. Prediction models for enzyme activity vs. temperature were determined at 70–85 °C, the range of best response (Figure 4). The best R^2^ coefficients were obtained with polynomic (>0.94 except for PG/RF) and exponential (>0.92 except for PPO/CH and PG/RF) second order equations. As pasteurisation temperature increased in the 70–85 °C range, PME, POD and PPO activities showed a declining trend that can be predicted. These equations allow calculation of enzyme activities at lower temperature intervals.

### 3.2. Thermal Damage 

Data on physicochemical indexes related to thermal damage are shown in Table 2. CH and RF pasteurisation had similar thermal effects on the salmorejo treated at the same temperature, with some exceptions. HTST pasteurisation resulted in some decrease in ascorbic acid. After pasteurisation, the highest concentrations of this acid (10–11 mg 100 mL^−1^) corresponded to intermediate temperatures (75–90 °C). Ascorbic acid degraded more in RF than in CH salmorejo at 100 °C. TBARS values used to assess lipid oxidation were low (0.38–0.50 mg MDA Kg^−1^) and tended to increase with temperature. MDA concentration was lower in CH than RF salmorejo at 100 °C. The pH of salmorejo increased slightly with pasteurisation and then remained over 3.8 in CH and RF samples. L* and h* values respectively ranged from 55–60 and 58–61 CIE units in the pasteurised product and tended to decrease with temperature. This occurred because b* values (yellowness) increased more than a* values (redness) in CH and RF samples. Viscosity calculated including the initial stress (V1) ranged from 19.9–29.7 mPa s, while viscosity directly measured in sheared samples (V2) ranged from 0.8–1.2 Pa s. Differences between V1 and V2 units were due to measuring conditions. Both viscosity values tended to increase with temperature in CH and RF samples. The V2 value was lower in RF salmorejo at 85–90 °C. The values of deformation energy and hardness (consistency) ranged from 3.9–6.0 mJ and 0.26–0.25 N, respectively. Both texture values similarly tended to increase with temperature in CH and RF samples. 

### 3.3. Sensory Changes

HTST pasteurisation led to some changes in appearance (orangeness and smooth surface), odour/flavour (loss of fresh-like nuances, less general intensity and more homogeneity) and texture (more consistency) (Table 3). Once pasteurised, CH and RF salmorejo presented similar sensory properties when heated at the same temperature, with some exceptions. The increase in pasteurisation temperature enhanced most of the abovementioned changes. Orangeness intensity tended to increase with temperature, with the lowest (over 5.0 in a 10-point scale) and highest (over 6.9) scores at 70 °C and 100 °C, respectively. Smooth surface intensity was not different in samples from any treatment. Overall odour/flavour and fresh odour/flavour intensities clearly decreased with temperature, scoring from 7.5 to less than 5 and from 7.0 to less than 4, respectively. In contrast, the increase in pasteurisation temperature resulted in a rise in odour/flavour homogeneity (from 3.3 to 6.3). This was consistent with results for other specific attributes, whose changes in intensity were excessively weak (tomato flavour), tended to decrease with temperature (acid taste, tomato odour and vinegar odour/flavour) or were not affected by the temperature (garlic odour/flavour). As regards texture, thickness intensity tended to increase with temperature, when consistency was evaluated with the spoon (from 4 to more than 7.4), while mouth-feel intensity also tended to increase with temperature although to a lesser extent (from 3.1 to 5.6).

## 4. Discussion

HTST pasteurisation largely ensured the microbiological quality of salmorejo. All CH and RF treatments resulted in an adequate decimal reduction of total and sporulated mesophilic microorganisms in the pasteurised salmorejo. RF was somewhat more effective in inactivating spores perhaps due to its good thermal penetration. According to US Food and Drug Administration (FDA) specifications, a minimum temperature–time of 71.1 °C for 3 s is sufficient to achieve a 5-Log reduction of vegetative bacterial pathogens in acidic products (pH < 4) [45] and the HTST conditions used in the present study exceed these specifications. Reference values of total mesophilic bacteria (3.1 Log CFU mL^−1^), lactic acid bacteria (3.1 Log CFU mL^−1^) and moulds and yeasts (3.6 Log CFU mL^−1^) published for different commercial pasteurised gazpachos at the end of shelf life confirmed that mild pasteurisation would be sufficient in preventing proliferation of altering microbes in these types of tomato products [46]. HTST conditions of 74 °C for 30 s have been proposed by Kessler (2006) [47] to pasteurise tomato juice (pH = 4.05). The natural acidity of salmorejo (pH = 3.2), a product containing vinegar (1.5% *w*:*w*), together with rapid cooling before packing might help successful pasteurisation.

In contrast, HTST pasteurisation (either CH or RF) proved less efficient in inhibiting tomato enzymes. As seen, raw salmorejo showed strong enzyme activities, which might be enhanced by processing conditions. Grinding favours enzyme release by cell disintegration [7], atmospheric cutting introduces oxygen and can favour oxidase activities [48], while pre-heating at 50 °C can increase enzyme reaction rates in tomato products [49]. It was reported that spoilage microorganisms have lower heat resistance than enzymes responsible for undesirable changes in fruit homogenates [50]. Pasteurisation methods tested in tomato products involve more intense heating in comparison to HTST salmorejo. In tomato purée, MW (390–3150 W; 150–848 s) heating was as efficient as CH (96 °C; 35 s); in inactivating PME and POD (12–16% and 15–17%, respectively), low MW (390–980 W; 340–848 s) reduced PG activity by half, while, when intensifying MW treatment, PG activity was reduced to 29% [9]. The same authors confirmed these findings in tomato smoothies [11]. In tomato juice treated with CH (69.8–77.8 °C; 200–1000 s), POD was less heat resistant than PME or PPO [51]. However, in tomato extracts, POD was slightly more resistant to CH (85–90 °C: 2 min) than PPO; when thermal treatment was prolonged to 12 min, there was a decrease in the %RE of POD (from 30–50% to 15–35%) and PPO (from 28–40% to 8–30%) [52]. In other studies, extending the duration of pasteurisation did not improve the inactivation levels reached for PME in tomato juice [12].

Tomato PG is considered a heat-resistant enzyme that requires high temperatures and long treatment times (e.g., 90–95 °C/30 min) to reduce its activity to a satisfactory level [4]. PG activity has in fact been used as an indicator of pasteurisation efficiency [11]. Temperatures higher than 90 °C for 5 min were needed to achieve complete inactivation of PG enzyme in tomato juice [37,53], therefore it is unlikely that this enzyme can be inactivated under HTST conditions. This does not mean that HTST salmorejo will undergo a quick clarification. PG and PME have synergistic activities, as demethylated pectin is the preferred substrate for the catalytic action of PG [54]. Thus, PG activity might be indirectly modulated through PME activity [37]. As seen, PME was easier to inactivate than PG in salmorejo. In a recent study, heating at 90 °C for 30 s practically inactivated the PME (10%) in a tomato juice [55]. Intensifying thermal treatment to inactivate the PG may be counterproductive for a “fresh-like” product such as salmorejo. As observed, PME, POD and PPO enzymes can be feasibly inhibited under HTST conditions in salmorejo. Based on results obtained for enzyme inactivation, the recommended temperature to pasteurise salmorejo was 80 °C (considering intervals of 5 °C). This temperature can be fitted using the regression equations obtained for each enzyme. At 80 °C, PME was completely inactivated, POD activity was reduced to 10%, PPO activity remained at around 33%, while, in contrast, PG activity was slightly enhanced. At 100 °C, the highest temperature tested, inactivation of POD or PPO did not improve, while PG activity was slightly inhibited. No data are available in the literature on the thermal behaviour of tomato enzymes under similar HTST conditions, although it was reported that PME and PG activities can decrease with the temperature in mild-heated tomato products (50–90 °C for up to 1 min) [12,37,51,54].

The increase in pasteurisation temperature led to some physicochemical and sensory signs of thermal damage in salmorejo, confirming that this product is sensitive to heat. Oxidation of ascorbic acid is considered as a direct indicator of thermal damage in fruit products [56]. As observed, salmorejo lost part of its ascorbic acid during pasteurisation. The best retention of ascorbic acid was reached at 80–90 °C, indicating that oxidase activities involved in its degradation were more efficiently inhibited, and also that thermal degradation of this acid was lower compared to the product treated at higher temperatures. Other studies agree that most vitamin C is retained by “fresh-like” tomato products, including salmorejo [3], gazpacho [57], juices [58], purées [9,59,60], smoothies [27] and vegetable beverages [13]. In contrast, pasteurised salmorejo only showed incipient MDA levels, therefore pasteurisation temperature was barely relevant for lipid oxidation. This suggests that the duration and/or temperatures used were poorly efficient in generating carbonyl compounds in the presence of lipid antioxidants such as oil olive tocopherols or tomato carotenoids. In fact, salmorejo did not develop rancid tones. Changes in odour and flavour due to heating mainly concerned the loss of freshness assessed in raw salmorejo. Heating also negatively affected the overall odour and flavour and favoured their homogenisation. Establishing prediction models of tomato odour/flavour descriptors based on the levels of volatile and non-volatile components is very complex since it involves a large number of compounds [61]. In the case of salmorejo, the contribution of other volatiles from olive oil, vinegar or garlic may complicate this task. Tomato contains fruity volatiles, such as terpenes, and others that are originated during processing by lipoxygenase activity, carotenoid co-oxidation and Maillard reaction that can be activated during the thermal treatments. The thermal treatment mainly modifies saturated and unsaturated C6 alcohols and aldehydes, esters, ketones and carotenoid derivatives [62]. Thermal treatment has a deleterious effect on green and fruity-note volatiles (e.g., hexanal) and generates cooked-note volatiles (e.g., dimethyl sulphide) that can be enhanced when temperature is intensified, leading to a decrease in perception of some appreciated odour and flavour attributes [63,64,65]. 

Colour is an important quality characteristic for pasteurised tomato products. Pasteurised salmorejo showed higher intensity of orange colour and smooth surface, which modify the original appearance of the raw product. Both chromatic and sensory data confirmed that salmorejo turned more orange when pasteurisation temperature was higher. Heat-treated salmorejo has been reported to have a more orange CIELAB colour than unpasteurised salmorejo [3]. Similar findings were reported for gazpacho [2], tomato smoothies [11], tomato juice [66] and tomato beverages [13] processed under HTST conditions. Colour degradation kinetics for salmorejo as a function of temperature shows some differences with respect to those reported for cooked tomato purée (50–120 °C for 15–30 min) [67]. Both salmorejo and tomato purée become darker (decrease in L*) due to heating; however, redness decreases in tomato purée (decrease in a*) but does not turn orange (increase in hue angle), as seen for salmorejo, likely due to the presence of olive oil. Heating leads to isomerisation of lycopene from the *trans* to *cis* form [68] and, since tomato redness depends on the level of trans-lycopene, severe thermal treatment leads to a decrease in redness [69]. 

Salmorejo became more consistent following heating, as confirmed by viscosity assessment. Consistency assessed with a spoon and, to a lesser extent, mouth-feel increased when using higher temperatures. The viscosity of tomato paste is related to the retention of pectin and destruction of PME and PG during thermal treatment. PME catalyses pectin de-esterification, and PG further catalyses the hydrolytic cleavage of the α-D-1,4-glycosidic bonds within the pectin acid chain. This causes depolymerisation of cell wall pectin when these two enzymes act synergistically, resulting in a decrease in viscosity of tomato products [37,70]. The time between crushing and hot breaking is also a crucial factor determining the degree of pectin destruction; the smaller the time gap, the higher the retention of pectin and the higher the viscosity [71]. Aguiló-Aguayo et al. (2008) [66] agree that an unheated tomato juice is less viscous than a pasteurised one (90 °C; 30 s). Salmorejo contains breadcrumbs rich in gelled starches with thickening properties not affected by tomato pectolytic enzymes. Moreover, the chicory inulin used to replace a part of the breadcrumbs is a purified ingredient without pectolytic enzymes, able to form thermal-reversible creamy gels by combining heating and shearing and can also increase salmorejo viscosity. 

**Figure 5:**
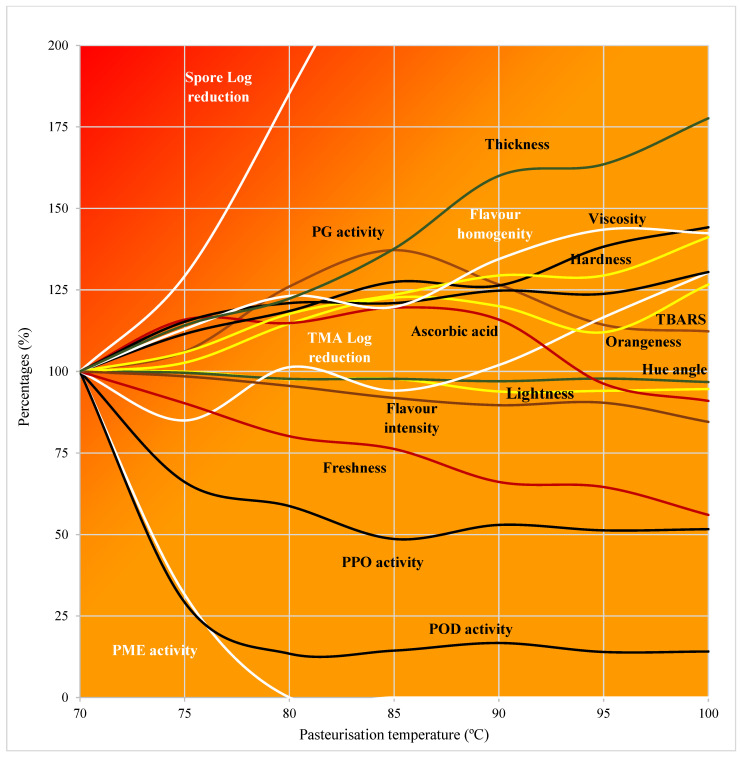
Relative effects of pasteurisation temperature (considered as 100% the values at 70 °C) on the quality traits of salmorejo.

Several studies agree that conventional and dielectric heating provide cooked and pasteurised tomato products of similar sensory quality. Desirable sensory attributes, for instance, colour, flavour and acceptability, were achieved in tomato purée cooked by CH (93.3 °C; 26.5 min) or MW (950 W; 3 min) [10], while Arjmandi et al. (2018) [27] reported slight differences in the appearance and flavour of tomato smoothies pasteurised by MW (210–3600 W; 93–646 s) or CH (90 °C; 35 s); in this study, the sensory quality of tomato smoothie slightly improved when using higher-power/shorter-time MW treatments. These data reveal that using conventional or dielectric HTST treatments is unlikely to produce marked sensory changes in fruit products, as with salmorejo. The lowest possible pasteurisation temperature should be chosen to guarantee microbiological quality and it should be assumed that salmorejo will maintain some remaining enzymatic activity. Intensifying the thermal treatment did not entail any technological advantage, since preservation levels barely improved and the risk of thermal damage to the product was higher (see Figure 5). Comparison between CH and RF raises some doubts about the interest in replacing conventional heat exchangers with RF equipment in the HTST pasteurisation lines for tomato products. Both technologies provided similar results in terms of the desirable effects of pasteurisation—maximum preservative efficiency with least thermal damage. This was likely due to the operating conditions applied, which were designed to be implemented in the industry. Pre-heating uses a part of the heat generated by cooling down the product, facilitates homogenisation and allows the pasteuriser to overcome the temperature ramp in a few seconds, as required for HTST processes. The flow rates and holding times are determined by the elements of each pasteuriser (flow rate of pump and design of heat exchangers). As seen, dielectric continuous heating in HTST conditions barely improved pasteurisation results in a viscous liquid food with laminar flow, such as salmorejo.

## 5. Conclusions

Salmorejo is prone to thermal damage in HTST conditions and requires careful pasteurisation. The main heat-induced changes include: orangeness, loss of freshness, thickening, flavour homogenisation, loss of vitamin C and lipid oxidation. Both CH and RF equivalent treatments (at the same pasteurisation temperature) allow a strong reduction of total and sporulated mesophilic microorganisms and an adequate inhibition of the PME, POD and, to a lesser extent, PPO enzymes but not of the PG enzyme. Pasteurisation at 80 °C for 9.8 s (RF) or 10.9 s (CH) provided a good equilibrium between preservation levels and thermal damage to the product. Increasing this temperature does not improve enzyme inactivation levels and salmorejo may become overheated. Assessment of thermal damage through physicochemical indexes (dynamic viscosity, texture, CIELAB colour, ascorbic acid and lipid oxidation) and quantitative sensory analysis confirmed this finding. A “fresh-like” good-quality salmorejo can be obtained using either conventional or radiofrequency continuous pasteurisers. Holding times and flow rates must be adjusted in each equipment. A further study will determine the factors (microbiological, physicochemical or sensory) that limit the shelf life of pasteurised (at 80 °C) salmorejo kept under refrigeration.

## Figures and Tables

**Table 1:** Definition of sensory attributes included in the sensory profile.

Attributes ^1^	Definition
Appearance	
Orange colour	Intensity of orange colour
Smooth surface	Intensity of homogeneous and soft surface
Odour/flavour	
Intensity	Intensity of overall odour/flavour
Tomato	Intensity of tomato odour/flavour
Garlic	Intensity of garlic odour/flavour
Vinegar	Intensity of vinegar odour/flavour
Homogeneity	Odour in which no attribute stands out
Fresh	Intensity of odour/flavour which corresponds to a freshly made product
Acidity	Basic taste sensation elicited by citric acid
Non-oral texture	
Viscosity with spoon	Difficulty with which salmorejo flows using a teaspoon
Oral texture	
Mouth-feel	Degree to which juice is thick, coats the mouth and difficult to swallow

^1^: Attributes were scored from 0 (lowest intensity) to 10 (maximum intensity).

**Table 2:** Physicochemical oxidation indexes determined in salmorejo pasteurised by convectional (CH) or radiofrequency (RF) heating at different temperatures (70–100 °C).

Temperature °C		Raw	70	75	80	85	90	95	100	
		M	M	M	M	M	M	M	M	RMSE
pH	CH	3.60	3.86 ^a^	3.82 ^ab^	3.82 ^ab^	3.79 ^b^	3.85 ^a^	3.85 ^a^	3.83 ^a^	0.01
	RF	3.59	3.83 ^ab^	3.80 ^ab^	3.80 ^ab^	3.80 ^ab^	3.79 ^b^	3.82 ^ab^	3.79 ^b^	
D-ascorbic acid	CH	12.2	9.2 ^b^	10.7 ^a^	11.3 ^a^	11.7 ^a^	10.1 ^a^	8.4 ^b^	9.7 ^b^	0.12
mg 100 mL^−1^	RF	14.8	9.7 ^b^	11.2 ^a^	10.4 ^a^	10.9 ^a^	11.9 ^a^	9.8 ^ab^	7.5 ^c^	
TBARS	CH	0.37	0.38 ^b^	0.39 ^b^	0.43 ^ab^	0.46 ^ab^	0.45 ^ab^	0.42 ^ab^	0.51 ^a^	0.01
mg MDA Kg^−1^	RF	0.33	0.37 ^b^	0.38 ^b^	0.43 ^ab^	0.46 ^ab^	0.45 ^ab^	0.42 ^ab^	0.44 ^ab^	
Lightness	CH	47.2	58.5 ^a^	57.9 ^ab^	56.4 ^ab^	56.6 ^ab^	55.0 ^b^	55.8 ^b^	55.6 ^b^	0.50
CIE L*	RF	48.1	59.3 ^a^	59.6 ^a^	58.4 ^a^	58.3 ^a^	55.3 ^b^	54.8 ^b^	55.7 ^b^	
Redness	CH	21.2	19.4 ^c^	20.6 ^bc^	22.1 ^ab^	22.2 ^ab^	22.5 ^ab^	22.6 ^ab^	23.7 ^a^	0.23
CIE a*	RF	19.9	19.4 ^c^	19.3 ^c^	20.3 ^bc^	20.8 ^bc^	21.9 ^ab^	22.0 ^ab^	21.8 ^ab^	
Yellowness	CH	24.9	34.1 ^b^	35.7 ^ab^	37.4 ^a^	37.6 ^a^	37.3 ^a^	37.6 ^a^	37.4 ^a^	0.64
CIE b*	RF	22.5	35.0 ^ab^	34.5 ^ab^	34.7 ^ab^	35.0 ^ab^	36.2 ^ab^	36.9 ^ab^	37.4 ^a^	
Hue angle	CH	32.7	60.4 ^a^	60.1 ^ab^	59.4 ^ab^	59.4 ^ab^	58.9 ^ab^	59.7 ^ab^	57.7 ^b^	0.80
CIE h*	RF	30.0	61.0 ^a^	60.8 ^a^	59.3 ^ab^	59.3 ^ab^	58.9 ^ab^	59.1 ^ab^	59.8 ^ab^	
Viscosity 1	CH	^(1)^	20.9 ^b^	23.3 ^b^	23.0 ^b^	25.5 ^ab^	25.2 ^ab^	27.3 ^a^	29.7 ^a^	1.93
mPa s	RF	^(1)^	19.9 ^b^	23.1 ^b^	22.6 ^b^	24.1 ^b^	22.0 ^b^	28.6 ^a^	28.8 ^a^	
Viscosity 2	CH	^(1)^	0.82 ^c^	0.86 ^bc^	1.08 ^ab^	1.16 ^a^	1.18 ^a^	1.19 ^a^	1.20 ^a^	0.02
Pa s	RF	^(1)^	0.80 ^c^	0.91 ^b^	0.95 ^b^	1.00 ^b^	1.04 ^b^	1.07 ^a^	1.15 ^a^	
Deformation	CH	^(1)^	4.1 ^d^	4.3 ^d^	4.6 ^cd^	5.0 ^bc^	5.2 ^b^	5.1 ^b^	5.7 ^a^	0.10
Energy mJ	RF	^(1)^	3.9 ^d^	4.0 ^d^	4.5 ^cd^	4.8 ^bc^	5.2 ^b^	5.1 ^b^	6.0 ^a^	
Hardness	CH	^(1)^	0.18 ^c^	0.19 ^bc^	0.21 ^ab^	0.21 ^ab^	0.21 ^ab^	0.21 ^ab^	0.23 ^a^	0.00
N	RF	^(1)^	0.16 ^c^	0.17 ^c^	0.19 ^bc^	0.21 ^ab^	0.23 ^a^	0.23 ^a^	0.25 ^a^	

^(1)^ Viscosity and texture could not be measured in raw and pasteurised samples using the same operating conditions. ^a–d^ Effects of heating method and temperature for *p* < 0.05 (Tukey test). There was interaction (*p* < 0.05) between treatments for TBARSs, L*, b*, h* deformation energy and hardness.

**Table 3:** Sensory changes determined in salmorejo pasteurised by convectional (CH) or radiofrequency (RF) heating at different temperatures (70–100 °C).

Temperature °C		Raw	70	75	80	85	90	95	100	
		M	M	M	M	M	M	M	M	RMSE
Orangeness	CH	4.4	5.5 ^b^	6.1 ^ab^	6.4 ^ab^	6.4 ^ab^	6.5 ^ab^	6.7 ^ab^	6.8 ^a^	0.31
	RF	4.2	5.0 ^c^	6.0 ^ab^	6.3 ^ab^	6.3 ^ab^	6.6 ^ab^	6.4 ^ab^	7.0 ^a^	
Smooth Surface	CH	2.7	6.8	6.7	6.5	6.1	6.5	5.8	5.5	0.64
	RF	3.0	6.9	6.9	6.6	6.4	6,4	6.3	6.0	
Odour Intensity	CH	6.9	6.6 ^a^	6.0 ^a^	5.6 ^a^	5.7 ^a^	5.7 ^ab^	5.4 ^ab^	5.0 ^b^	0.38
	RF	7.0	6.5 ^a^	6.2 ^a^	5.7 ^a^	5.8 ^a^	5.4 ^ab^	5.5 ^ab^	5.1 ^b^	
Flavour Intensity	CH	7.1	6.7 ^a^	6.8 ^ab^	6.3 ^bc^	6.2 ^bc^	5.9 ^bc^	6.0 ^bc^	5.5 ^c^	0.56
	RF	7.7	7.3 ^a^	6.6 ^ab^	6.7 ^ab^	6.3 ^bc^	6.3 ^bc^	6.3 ^bc^	6.0 ^bc^	
Odour Homogeneity	CH	3.3	4.3 ^b^	4.9 ^b^	5.4 ^ab^	5.8 ^ab^	6.1 ^a^	6.6 ^a^	6.4 ^a^	0.47
	RF	3.3	4.8 ^b^	5.1 ^b^	5.5 ^ab^	5.8 ^ab^	5.8 ^ab^	6.1 ^a^	6.2 ^a^	
Flavour Homogeneity	CH	3.3	4.3 ^b^	4.9 ^b^	5.4 ^ab^	5.8 ^ab^	6.1 ^a^	6.6 ^a^	6.4 ^a^	0.47
	RF	3.3	4.8 ^b^	5.1 ^b^	5.5 ^ab^	5.7 ^ab^	5.8 ^ab^	6.1 ^b^	6.2 ^a^	
Fresh Odour	CH	6.7	6.4 ^a^	5.5 ^b^	4.7 ^c^	4.7 ^cd^	4.2 ^de^	3.8 ^de^	3.6 ^e^	0.56
	RF	6.8	6.6 ^a^	5.7 ^b^	5.0 ^c^	4.8 ^cd^	4.9 ^cd^	4.3 ^de^	4.3 ^de^	
Fresh Flavour	CH	6.9	6.4 ^a^	5.6 ^b^	5.0 ^c^	4.8 ^cd^	4.1 ^d^	3.8 ^d^	3.3 ^de^	0.69
	RF	7.2	6.5 ^a^	6.0 ^ab^	5.3 ^c^	5.0 ^c^	4.4 ^cd^	4.5 ^cd^	3.9 ^d^	
Tomato Odour	CH	6.3	4.5 ^a^	3.9 ^ab^	3.6 ^bc^	3.8 ^bc^	3.7 ^bc^	3.5 ^c^	3.2 ^c^	0.36
	RF	6.2	4.2 ^b^	4.1 ^b^	3.9 ^ab^	3.8 ^bc^	3.7 ^bc^	3.8 ^bc^	3.5 ^c^	
Vinegar Odour	CH	5.1	4.4 ^a^	4.6 ^a^	3.9 ^ab^	3.9 ^ab^	3.6 ^ab^	3.6 ^ab^	3.1 ^b^	0.59
	RF	4.9	4.8 ^a^	4.4 ^a^	4.0 ^ab^	4.1 ^ab^	3.8 ^ab^	3.4 ^ab^	3.3 ^b^	
Vinegar Flavour	CH	5.4	5.5 ^a^	4.6 ^a^	4.5 ^a^	4.4 ^ab^	3.6 ^bc^	3.5 ^bc^	3.2 ^c^	0.30
	RF	4.9	5.0 ^a^	4.4 ^a^	4.4 ^a^	4.4 ^ab^	3.7 ^bc^	3.8 ^bc^	3.4 ^c^	
Garlic Odour	CH	5.0	3.3	3.5	3.3	3.4	3.1	3.2	3.1	0.56
	RF	5.0	3.5	3.5	3.2	3.4	3.4	3.2	3.0	
Garlic Flavour	CH	4.3	4.2	3.9	3.7	3.6	3.7	3.6	3.5	0.83
	RF	4.4	4.3	3.9	3.6	3.6	3.4	3.2	3.5	
Acidity	CH	5.2	5.4 ^a^	4.8 ^a^	4.2 ^ab^	4.2 ^ab^	4.1 ^ab^	4.0 ^ab^	3.8 ^b^	0.32
	RF	5.3	5.0 ^a^	4.7 ^a^	4.4 ^ab^	4.4 ^ab^	4.3 ^ab^	4.5 ^ab^	4.1 ^b^	
Spoon Consistency	CH	3.8	4.1 ^d^	5.0 ^d^	5.2 ^cd^	6.1 ^c^	6.9 ^ab^	7.0 ^ab^	7.7 ^a^	0.30
	RF	4.1	4.4 ^d^	4.7 ^d^	5.2 ^cd^	5.6 ^c^	6.7 ^ab^	6.9 ^ab^	7.4 ^a^	
Mouth-feel	CH	3.2	3.4 ^d^	3.6 ^d^	3.9 ^cd^	4.3 ^c^	4.6 ^ab^	5.2 ^ab^	5.5 ^a^	1.27
	RF	3.1	3.0 ^d^	3.5 ^d^	3.7 ^cd^	3.8 ^c^	4.7 ^ab^	4.6 ^ab^	5.7 ^a^	

Abbreviations: M: mean; RMSE: root mean standard error. Units: 0–10 intensity scale. ^a–e^ Effects of heating method and temperature for *p* < 0.05 (Tukey test).

## Data Availability

The datasets generated and/or analysed during the current study are available from the corresponding author upon reasonable request.
